# Frequency-Domain sEMG Classification Using a Single Sensor

**DOI:** 10.3390/s22051939

**Published:** 2022-03-02

**Authors:** Thekla Stefanou, David Guiraud, Charles Fattal, Christine Azevedo-Coste, Lucas Fonseca

**Affiliations:** 1 Camin Team, National Institute for Research in Computer Science and Automation (Inria), 34090 Montpellier, France; david.guiraud@inria.fr (D.G.); cfattal@ussap.fr (C.F.); christine.azevedo@inria.fr (C.A.-C.); lucas.fonseca@inria.fr (L.F.); 2 Neurinnov, 34600 Les Aires, France; 3 Rehabilitation Center Bouffard Vercelli, USSAP, 66000 Perpignan, France

**Keywords:** assistive technologies, EMG, classification, frequency domain, intent detection, prosthesis

## Abstract

Working towards the development of robust motion recognition systems for assistive technology control, the widespread approach has been to use a plethora of, often times, multi-modal sensors. In this paper, we develop single-sensor motion recognition systems. Utilising the peripheral nature of surface electromyography (sEMG) data acquisition, we optimise the information extracted from sEMG sensors. This allows the reduction in sEMG sensors or provision of contingencies in a system with redundancies. In particular, we process the sEMG readings captured at the trapezius descendens and platysma muscles. We demonstrate that sEMG readings captured at one muscle contain distinct information on movements or contractions of other agonists. We used the trapezius and platysma muscle sEMG data captured in able-bodied participants and participants with tetraplegia to classify shoulder movements and platysma contractions using white-box supervised learning algorithms. Using the trapezius sensor, *shoulder raise* is classified with an accuracy of 99%. Implementing subject-specific multi-class classification, *shoulder raise*, *shoulder forward* and *shoulder backward* are classified with a 94% accuracy amongst *object raise* and *shoulder raise-and-hold* data in able bodied adults. A three-way classification of the platysma sensor data captured with participants with tetraplegia achieves a 95% accuracy on *platysma contraction* and *shoulder raise* detection.

## 1. Introduction

Human–machine interfaces (HMI) are a crucial part of assistive and rehabilitation devices such as prosthetic limbs, exoskeletons or neuroprosthetic solutions. Indeed, it is essential to correctly identify the user’s intent or orders to properly control such devices. It has been shown that useful information can be extracted by employing various modes of communication [1,2]. To activate an assistive device, a system may employ speech recognition [3], eye tracking [4,5], kinematic data [6,7], push-buttons [8] or biological signals such as electromyography [9,10]. A number of systems employ multimodal strategies, such as measurements of muscle activation in parallel with joint kinematics [11,12,13]. This provides diverse information for the decision-making algorithm and control input. However, there is a large gap between the academic state-of-the-art systems and the commercially available ones. There has so far been little improvement in functionality, intuitiveness and performance in those solutions when used in everyday life. The structured environments in which the majority of the solutions proposed throughout the years have been evaluated fall short in replicating real-life challenges [14]. The classification accuracies and the sophistication of the methods used have not yet translated to the real world, while their practicality and calibration needs are unrealistic.

In the present study, we analysed surface electromyography (sEMG) signals of specific muscles that will be used to activate an upper-limb neuroprosthesis developed for individuals with complete tetraplegia (lesions at C4–C6). Surface EMG recordings are peripheral measurements that contain neural information on the activation of muscles in the vicinity of the sensor(s) and thus indirectly on the motor task performed.

We use white-box algorithms to find a compromise between parsimony (least possible number of inputs/sensors) and robustness to develop systems which are independently set up and can be used outside the lab. Taking advantage of the peripheral nature of sEMG data, we demonstrate how one can minimise the number of required EMG channels (or provide contingencies in a system that can now be thought of as redundant) while ensuring robustness to obtain an easy-to-use EMG system that is able to detect several intents from the participant. The use of white-box algorithms is of significance due to their transparency and thus inherent safety. Furthermore, we initiate the discussion as to why it is feasible to perform multi-class classification using a single sensor for movements of different joints or with different agonists and how, what would otherwise have been considered noise, can be used to our advantage.

Selecting the appropriate number of EMG channels and the positioning of the electrodes is an important aspect in sEMG-based interfaces [15]. The number of channels relates directly to the number of muscles from which activity has to be monitored. The optimal number and distribution of electrodes has been studied as a factor of classification accuracy. In multiple studies on the control of upper limb exoskeletons and prostheses, the majority of the systems have used eight channels on the shoulder, the forearm or upper arm [16,17]. However, four to six bipolar electrodes were found to be sufficient at recognising various hand motions [18] even though it also depends on the anatomy of the subject [19]. For small subsets of data, incrementing the number of channels was found to improve the accuracy when the channels were placed further away from each other; this is a result of the capture of new information from muscle activity [20]. However, the failure of one electrode in these cases could cause significant degradation in the classification accuracy.

High-density surface EMG systems (HDsEMG), including recently developed miniaturised HDsEMG systems [21], have been used to detect motor unit (MU) activity [22,23] and decompose it into motor unit spike trains (MUSTs). Decomposition techniques have been used to map MUSTs to continuous kinematics of hand movements [24,25]; high-dimensionality EMG data is factorised into low-dimensionality control signals. Hug et al. [23] looked at the reliability of such decompositions and proved their consistency, demonstrating that they are a reliable technique for the extraction of upper-limb kinematics. A lot of past studies monitored muscular activity in the forearm that embeds about twenty different muscles. Che et al. used 2 grids of 64 channels on the forearm to recognise wrist and grasp movements [26]. Such methodologies have been used to analyse muscle synergies [27,28] achieving a real-time task completion rate of 95% [29]. Using HDsEMG solves some of the problems of sEMG; finding the best electrode placement for optimal operation is no longer necessary, whereas the effects of any positional shifting of the electrodes that may happen are mitigated. However, today, HDsEMG remains costly and cumbersome in real-life applications and has high computational requirements.

Studies have so far focused on systems with multiple control inputs; this raises the complexity and required processing power. The amount of information that can be extracted from a single sEMG signal has not yet been fully explored. Maximising the information from one sensor can provide fail-safes in a motion intent recognition system that depends on multiple sensors and can lead to more robustness and a simplified donning. Furthermore, this approach turns what could be thought of as noise or crosstalk into a solution rather than a problem to be overcome. To our knowledge, single-channel EMG control systems have not been explored very extensively. An exception is a study by Tavakoli et al. [30] where a support vector machine classifier was used on sEMG data from one bipolar electrode to distinguish between hand opening, hand closing and wrist flexion. The beginner users achieved lower accuracies (81%) than the experienced users (94%), but with training, the accuracy increased to at least 90%. A two-sensor system was later used by the same authors to classify five hand gestures with a classification accuracy between 95% and 100% [31]. In addition, Hameed et al. [32] determined that the best muscle to place a single sEMG sensor on the forearm in order to classify low signal-to-noise ratio hand activities is the flexor carpi ulnaris.

Our hypothesis is that a single sEMG recording contains enough information to detect several movements. Indeed, even though a single muscle’s activity is targeted, we see here that its activity has a specific signature depending on the movements performed. At the same time, other proximal muscles’ activities are partly reflected in the sEMG signal. We thus try to promote a more optimal use of sEMG signal to better fit a future “home use” system. The question we want to answer is how much neural information can be acquired from a single sEMG sensor even in the presence of noise? To what extent can we distinguish between different movements with information from one single muscle? Are there features in the signal that could indicate the engagement of a second muscle?

In this paper, we look at the sEMG signal’s frequency domain features and, in particular, the power spectral density (PSD). The PSD has been shown to contain useful information when comparing neuropathic and healthy subjects’ EMG signals [33] and it has long been used to evaluate muscle fatigue [27,34]. Other studies [35] have demonstrated that spontaneous or involuntary EMG activity is likely to be present in background noise and thus would not be easily visible in the time domain but could be distinguished in the frequency domain using PSD and multi-scale entropy. We thus hypothesised that a relevant signal processing framework from which to extract meaningful information would be the frequency domain.

The main aim of this work is to demonstrate how one can classify different agonist and joint actions using sEMG data from a single sensor, working towards the development of real-world systems. The proposed methods were able to correctly classify three shoulder actions and shoulder movement and platysma contraction even in the presence of noise; which was the case in the data of individuals with tetraplegia. Thus the system proposed is viable as a user interface for assistive devices.

## 2. Materials and Methods

This study includes data from two protocols: one involves 10 able-bodied adults (5 men, 5 women) and the other 2 male participants with complete tetraplegia (AIS A, C4 level). This was performed within the scope of the AGILIS project (Restoration of hand Functions in Tetraplegia through Selective Neural Electrical Stimulation, https://eithealth.eu/project/agilis/—accessed 25 January 2022). In the study with able-bodied participants, all participants signed the written consent and the protocol was approved by Inria’s Operational Committee for the assessment of Legal and Ethical risks (COERLE) on 28 July 2021, authorisation number #2021-28. During the study with participants with tetraplegia, the participants provided written informed consent before participating, in accordance with the Declaration of Helsinki. The protocol was approved by the Ethics Committee in 2020 (CPP Ouest IV Nantes, France, ID-RCB #2019-A02037-50) and the Health Agency (ANSM). The study was registered on ClinicalTrials.gov (NCT04306328).

### 2.1. Protocol A: Able-Bodied Participants

**Figure 1:**
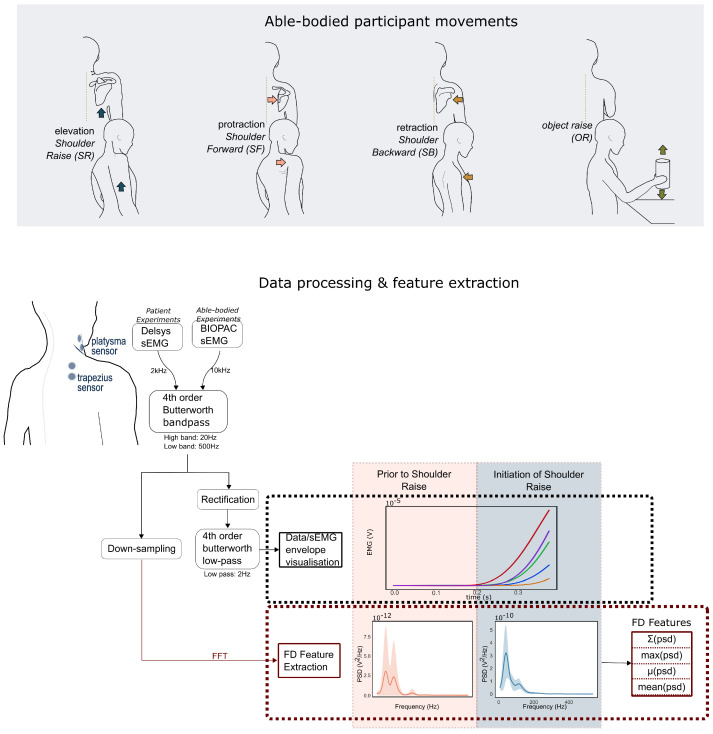
(**Top**): The movements attempted by the able-bodied participants in Protocol A (Section 2.1). (**Bottom**): The flow diagram demonstrates the processing of the data leading to the extraction of the frequency domain data and visualisation of the signal’s envelope. Five instances of shoulder raise are presented in the time and frequency domains, before and after the action. In the frequency domain plots, the bold lines present the mean PSD and the shaded regions the standard deviation.

The experiments with able-bodied individuals aimed to demonstrate that we can differentiate various shoulder movements based on different muscle activation patterns using a minimal set of sEMG sensors and frequency-domain features. The data were captured using the EMG100C (BIOPAC Systems, Goleta, MA, USA) with the NI USB-6218 16-bit ADC (National Instruments, Austin, TX, USA) which has a 10 kHz sampling rate and a 3 kHz low-pass filter. One pair of EMG electrodes was placed on top of the upper trapezius muscle (trapezius descendens) following the SENIAM recommendations [36] with regards to placement and the distance between electrodes. The participants were asked to perform the following actions (shown in Figure 1):

- Basic shoulder movements for algorithm development: shoulder elevation, shoulder protraction and shoulder retraction—referred to as *shoulder raise* (*SR*), *shoulder forward* (*SF*) and *shoulder backward* (*SB*), respectively, in this paper. All of these were performed with a quick action-and-release.
- Additional movements used for evaluation: *shoulder raise-and-hold* (*SRH*) for 1 s, and *object raise* (*OR*), which consist of raising a water bottle (weighing 650 g) between shoulder and eyesight level and putting it back down (2 s).

For each task the instructions were provided on a screen; the participant was asked to relax (2 s), prepare (2 s) and then perform the action. In each trial, these instructions were repeated 10 times. Prior to the start of the experiment, the participants were instructed to perform the movements in a relaxed manner and vary the effort used to obtain a diversity of readings, simulating a real-world scenario, without exerting unnecessary effort.

### 2.2. Protocol B: Participants with Tetraplegia

The experiments with the spinal cord injury participants, with complete tetraplegia, aimed to demonstrate how the data analysis and frequency-domain feature classification performs in the presence of low signal-to-noise ratio and limited voluntary movement control and, most importantly, show how one sensor’s data can be used to recognise sEMG signal patterns from different sources/muscles. The Delsys sEMG system (Delsys, Natick, MA, USA) was used in these trials and the data acquisition rate was 2.15 kHz. For Participant1, who had limited control of his upper body, one sEMG sensor was placed on top of the upper trapezius muscle. For the experiments with Participant2, in addition to the upper trapezius muscle sensor, a second one was placed on the platysma muscle, a superficial neck muscle. Its positioning was determined by visually locating the belly of the muscle. During these trials the participants were verbally instructed to either raise their shoulder or contract their platysma muscle.

### 2.3. Pre-Processing

Raw sEMG signals have a frequency content between 6 Hz and 500 Hz with the highest spectral power being between 20 and 150 Hz. The signal was passed through a band-pass filter with cutoff frequencies at 20 Hz and 500 Hz (fourth order Butterworth) to obtain the raw band limited EMG data, further named EMG. Then, the signal was rectified, and a low pass filter (2 Hz, fourth order Butterworth) was applied to obtain the EMG envelope (Figure 1), further named eEMG. Subsequently, in the able-bodied subject trials, the data were down-sampled by a factor of 4 using an 80th order FIR filter.

### 2.4. Frequency Domain Analysis

Observing the data in the frequency domain (FD), and in particular the power spectral density (PSD) of the EMG signals, we can extract distinguishing features that allow us to classify the data. Indeed, the PSD of the signal, which shows the energy as a function of frequency, changes during each muscular activity (Figure 1). The maximum of the PSD waveform and the mean and median provide a good representation of the PSD waveform shape. The FD analysis was performed on the non-rectified data since rectification is only beneficial when assessing the strength of the low-frequency content (<12 Hz) [37]. The sEMG signals were used to visualise the signal’s envelope and compare the representations of the signal in the time and frequency domains.

Welch’s method was used to estimate the PSD since it reduces the noise in the power spectrum compared to the use of a standard periodogram. Unless otherwise stated, a sliding window of 0.2 s was used when estimating the PSD across the data. The window chosen was determined experimentally as it has to be large enough to fully capture the initiation of the movement but as small as possible to provide a quick classification. The spectogram was estimated using a Hann window, which provides a good resolution: 0.05 s segment lengths, with a 50% overlap, across which the average was calculated. The resulting spectrum had a resolution of 8 Hz.

### 2.5. Classification Method Selection

The frequency signatures of the sEMG signals were used to interpret neural information from the platysma and upper trapezius muscles. We used the features derived from the estimated spectograms to detect platysma and trapezius contractions in participants with tetraplegia and to differentiate between various motor tasks in the data of able-bodied participants. For the able-bodied individuals, decision trees were considered because the data can be described by attribute–value pairs. However, for the data of patients with tetraplegia, where there was a lower amount of data with higher variance and more labelling inaccuracies, decision trees were not suitable.

**Figure 2:**
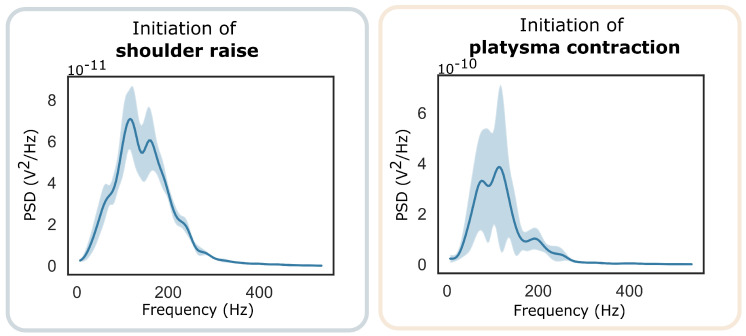
The average PSD of the platysma muscle sEMG signal around the points of contraction during five *shoulder raise* instances (**left**) and five *platysma contraction* (**right**) instances.

Participant1 with tetraplegia had very little strength and difficulty controlling his trapezius muscle. Participant2 was able to perform both shoulder raise and platysma contractions. His data were used to study the hypothesis that a single sEMG signal contains multiple separate, distinguishable events by analysing platysma sensor data. Figure 2 demonstrates how the power of the signal as well as information on the mean and median values of the PSD can be used to distinguish between the two states.

Despite the clear separability of the classes, cross-validation indicated that the decision tree algorithm could not perform well on the tetraplegic individuals’ data. This is probably due to the combination of the limited number of samples, the high variance in the data and labelling inaccuracies which are preventative when attempting to capture the diversity of these data. This was also evident in the inconsistency of the cross-validation results. We thus classified the data by thresholding the same FD features as the ones used by the decision tree (Section 2.5.2).

#### 2.5.1. Decision Trees

A decision tree classifier is a supervised, non-parametric white-box algorithm that uses a tree structure where each node tests an attribute and each branch corresponds to an attribute value stemming from said node, and the classification is assigned at the end leaf nodes [38]. Decision trees are intuitive, fast and efficient with low-effort training. Most importantly, they provide transparency; due to the white-box nature of such algorithms, we know how predictions are made and what influences them and how, which makes them inherently safer to use. Such an algorithm relies on the training data quality and its accuracy decreases around decision boundaries. Noisy instances, a small number of training examples and over-learning are some of the main reasons that could lead to poor performance [39] and overfitting.

##### Parameters

Applying the CART algorithm on the data, the decision tree that models the system is formed. The “best” split at each node was determined using the Gini impurity index, finding the features that minimise error at each branch/node. At each stage (node) of the decision tree, the features are considered and a condition is set to split the data. The feature that offers the lowest impurity score is the one chosen as the algorithm aims to make every node as homogeneous as possible using the Gini impurity equation;

(1) $$G=1-\sum_{c=1}^{n}p_{ic}^{2}$$

where *n* is the total number of classes and *p* is the probability that a particular sample in that node belongs in class *c*. The lower the value of *G*, the higher the purity in the node.

After shuffling the data, weights were applied to balance the classes since there are disproportionately more *no action*-labelled class datasets than any other. This purposely modifies the loss function and biases the model to favour more accurate predictions of the higher-weighted, minority classes. Thus, the classifier learns equally from all classes.

Pre-pruning was used to stop the classifier from overfitting on the training data; this early stopping heuristic stops the development of the tree prematurely to satisfy certain parameter limits. We initially performed a grid search of the three hyperparameters that control the pre-pruning, the maximum depth of the tree, the minimum number of data samples at each leaf node and the minimum number of samples required at each node, before manually tuning them for each participant accordingly to avoid overfitting. The grid search indicated the parameter values that resulted in the best classification accuracy. Observing the confusion matrices, the parameters were tuned for each participant individually to optimise the prediction accuracy across all classes.

##### Train/Test Splits

For each training and testing of the decision tree model, a 10-fold cross-validation was implemented using a stratified split; this ensured that the classes were equally split amongst the 10 groups of data. For each participant, there were about 150 k data for training per trial/action. Initially, we looked at the detection of *shoulder raise* which is the movement used for the activation of the neuroprosthesis and whose primary agonist is the upper trapezius muscle. The *SR*, *SF* and *SB* data were split into training and test datasets and at each iteration the model was further tested using the unknown data of *SRH* and *OR* actions. Following that, we performed a four-way classification of the three basic shoulder movements using the *SR*, *SF* and *SB* data. Just like in the binary classification, at each training/testing loop, the model was also tested against the SRH and *OR* data.

#### 2.5.2. Thresholding Algorithm

To implement a thresholding algorithm, the data were fragmented into *action* and *no action* windows and labelled accordingly. Audiovisual recordings showing when the instruction was given and an indication of when the shoulder raise or platysma contraction attempts may have occurred were used to set the ground truth. To calculate the accuracy of the system, the data points were grouped as follows. We defined an *action window* which was twice as long as the time frame of the action performed beginning when an instruction is given; all data points within this window were grouped together and each group was labelled accordingly. The *action windows* were then split into ten stratified groups, such that each class was equally represented in each. During each training cycle, one group was held out for testing and among the rest of the data the features were calculated. For each instance of an action, the maximum feature values were extracted and, using those, the features’ medians were estimated for each action. These were then used to set the thresholds for the class separation and tested against the *no action* and the remaining *action* data. For binary classification, all four thresholds have to be satisfied to determine that a specific action has been performed. For multi-class classification, the third quartile value was estimated for each feature and used as the maximum threshold for a class. To measure the accuracy of the system, all predictions within each *action* and *no action* window were considered. If the wrong action was predicted anywhere within an action window, the window as whole was classified as such. On the other hand, if the correct action was detected within the action window, and no wrong action was detected, then that was deemed a correct classification.

The classification algorithms presented were implemented to binary and multi-class classification on both able-bodied and tetraplegic individuals’ data. The results and their significance are presented below.

## 3. Results

This section presents the results of binary classification when predicting *shoulder raise* actions in able-bodied and tetraplegic individuals. Following that, multi-class classification is attempted where the data from one sEMG sensor are used to detect multiple actions. This leads up to the classification of both shoulder raise (SR) and platysma contraction (PC) in a participant with tetraplegia using the sensor placed on the platysma muscle. The number of data classified for each individual are presented in Table 1. Each sample represents a 0.2 s window.

### 3.1. Binary Classification: Shoulder Raise

#### 3.1.1. Able-Bodied Participants: SR

**Figure 3:**
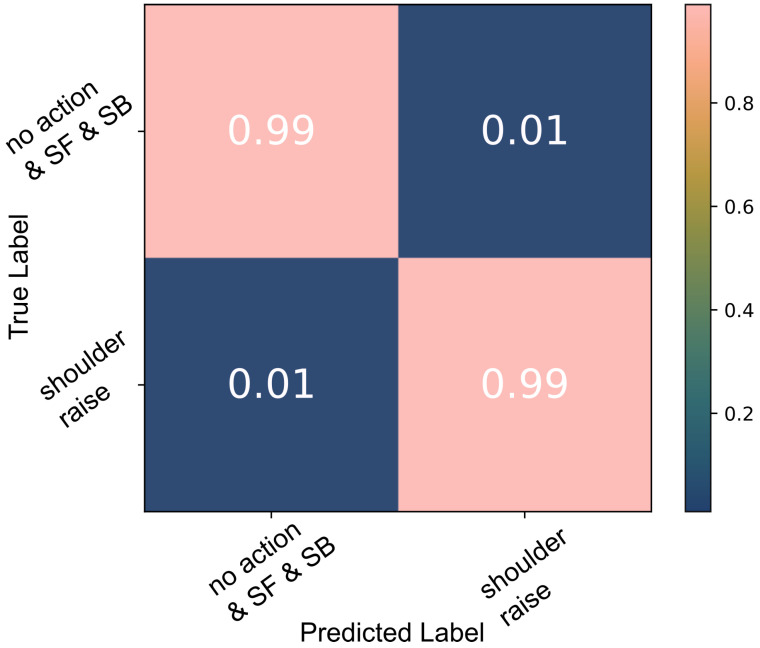
The decision tree classification results of the able-bodied participants’ *SR* data; the model was tested against *SR*, *SF* and *SB* data.

**Figure 4:**
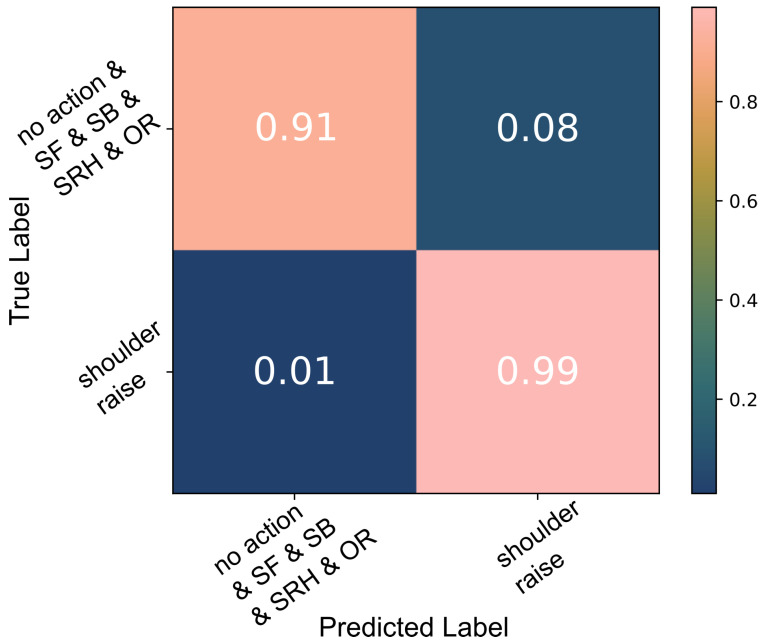
Decision tree classification results of able-bodied participants’ *SR* data; the model was tested against all data including the unknown *SRH* and *OR* action data.

A decision tree was trained to detect *SR* on the data captured during the three basic shoulder movements performed by the able-bodied individuals. A 10-fold cross-validation indicated a 99% accuracy as shown in Figure 3. Each time the model was trained and validated, it was also tested against the second group of unknown data, *shoulder raise-and-hold* (*SRH*) and *object raise* (OR), which were labelled as *no action*; 92% of all test data were classified correctly (Figure 4). When the algorithm was trained using all data available, the test accuracy was 99%.

**Figure 5:**
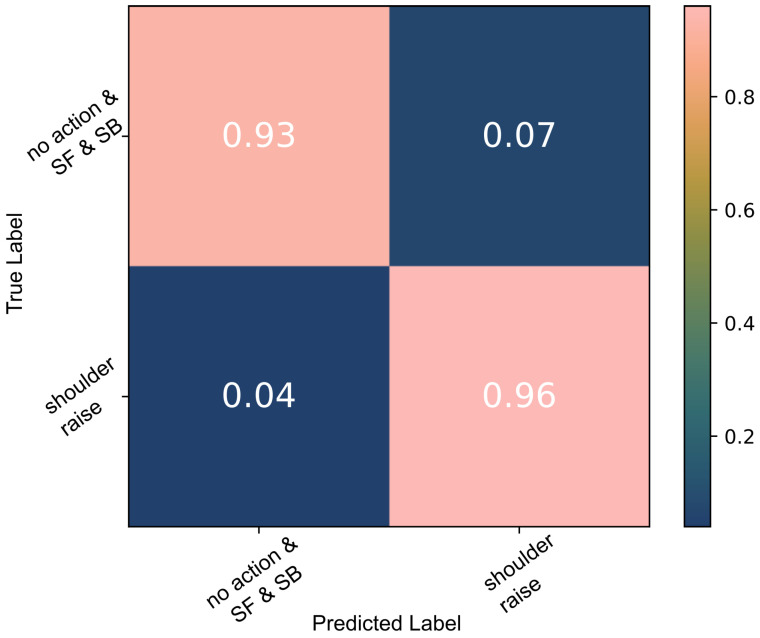
The thresholding algorithm *SR* classification results of able-bodied participant data; the model was tested against *SR*, *SF* and *SB* data.

**Figure 6:**
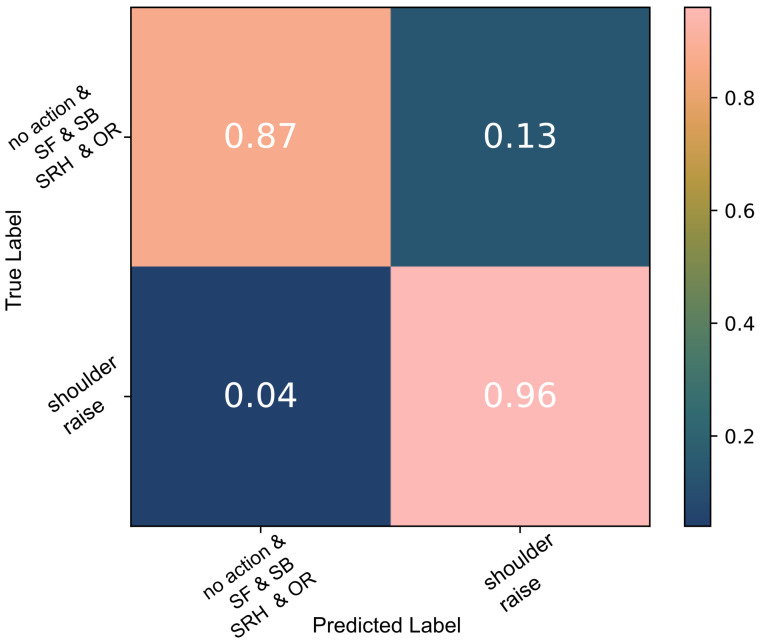
The thresholding algorithm *SR* classification results of able-bodied participant data; the model was tested against all data, including the unknown *SRH* and *OR* actions.

The same data were used to evaluate the thresholding algorithm and compare its performance to the one achieved using a decision tree. As described in detail in Section 2.5.2, the threshold applied by the algorithm is the median value of each feature during contraction; the cross-validation results of this classifier are shown in Figure 5 and Figure 6.

With the exception of two cases, all *SR* false positives seen in Figure 6 were instances of *SRH*. The thresholding algorithm did not perform as well as the decision tree algorithm. However, the results were on par with state-of-the-art [17] and thus the algorithm was used on the data of individuals with tetraplegia. Due to the lower sample numbers, high variance and labelling inaccuracies, the decision tree algorithm failed to capture their diversity.

#### 3.1.2. Individuals with Tetraplegia: SR & PC

**Figure 7:**
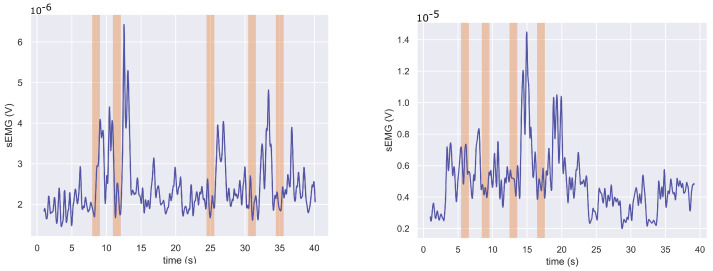
The sEMG envelope of data captured at the trapezius muscle of Participant1 during two trials (Trial1 on the **left** and Trial2 on the **right**).

**Figure 8:**
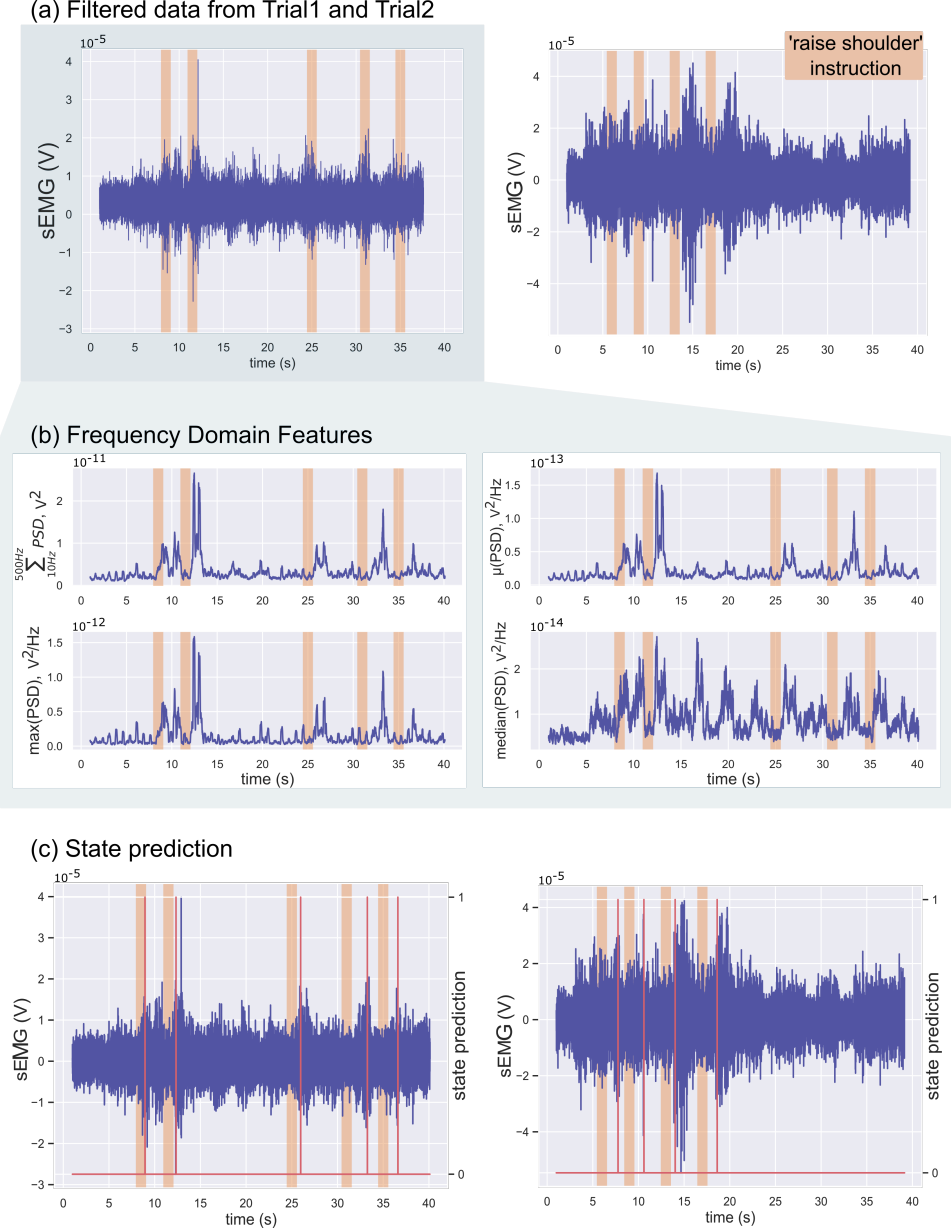
EMG data and classification for Participant1. (**a**) The filtered data from the trapezius muscle of the two Participant1 trials; Trial1 on the left and Trial2 on the right. (**b**) The four classification features from the Trial1 data. (**c**) The state prediction for the two trials.

Participant1 had very little strength and difficulty controlling his trapezius muscles. It was, therefore, challenging to distinguish the trapezius muscle activation from the noise in Participant1’s data even after filtering and rectifying the signal, as seen in Figure 7. The figure presents the filtered trapezius data recorded during the first (left plot) and second trials (right plot), the only two trials recorded (on two different days). The shaded regions in the figure indicate the instances when a verbal instruction was given during the trials. Figure 8 demonstrates the separability of the noisy data in the frequency domain features and how those were used to classify Participant1’s data. The data were split into five and four training/testing groups for Trial1 and Trial2, respectively, as those were the number of *SR* occurrences.

The data captured with Participant2 were used to classify *SR* and *PC* using a 5-fold cross-validation. The data were captured over the span of two days and the algorithm was run on the trials of each day individually. The trapezius sensor data were used to determine *SR* and all instances were correctly classified with no false positives. Following that, the thresholding algorithm also managed to correctly identify all but one platysma contraction.

Having shown how the algorithms perform on binary classification, we attempted to (a) classify three shoulder joint movements controlled by different muscles and (b) two different actions occurring at different parts of the body, controlled by different muscles using the data of a single sensor.

### 3.2. Multi-Class Classification

#### 3.2.1. Single Sensor, Three Shoulder Actions

**Figure 9:**
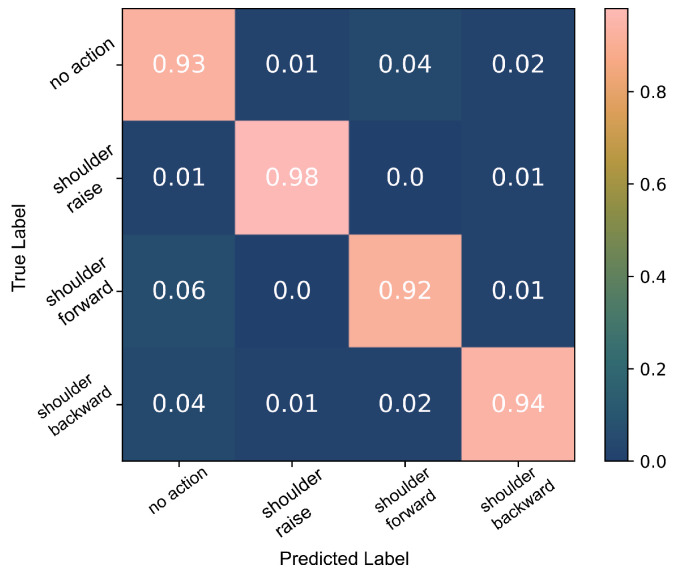
Classification of the three shoulder movements: the 10-fold cross-validation results of the decision tree algorithm which was trained using all able-bodied participants’ data.

A decision tree classifier was trained to detect the three shoulder movements, *SR*, *shoulder forward* (*SF*) and *shoulder backward* (*SB*), using all available data. The 10-fold cross-validation indicated that the model can classify the data with a 94% accuracy, Figure 9.

**Figure 10:**
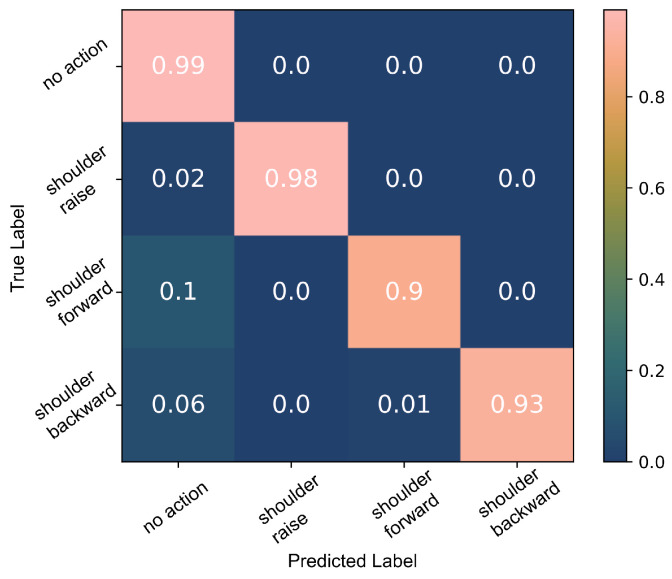
Classification of the three shoulder movements: the 10-fold cross-validation results of the decision tree algorithm which was trained using *SR*, *SF* and *SB* data.

The decision tree model was trained a second time using only the *SR*, *SF* and *SB* data. The 10-fold cross-validation results are presented in Figure 10. Testing the trained model against all data including *SRH* and *OR*, a 92% accuracy was calculated. The majority of the *no action* data misclassified as *SR*, 3% of the *no action* data, belonged to the *SRH* class. About 2% of *no action* data were incorrectly classified as *SF* and 2% as *SB*. These were largely a result of *OR* actions.

#### 3.2.2. Single Sensor, Neck and Shoulder Actions

As Participant2 was able to perform both shoulder raise and platysma contractions, his data were used to explore the hypothesis that a single sEMG signal contains separate, distinguishable events during each of those actions. Evidence of the *SR* events/trapezius muscle contractions are visible in the signal of the platysma muscle sensor both in the time and frequency domains.

**Figure 11:**
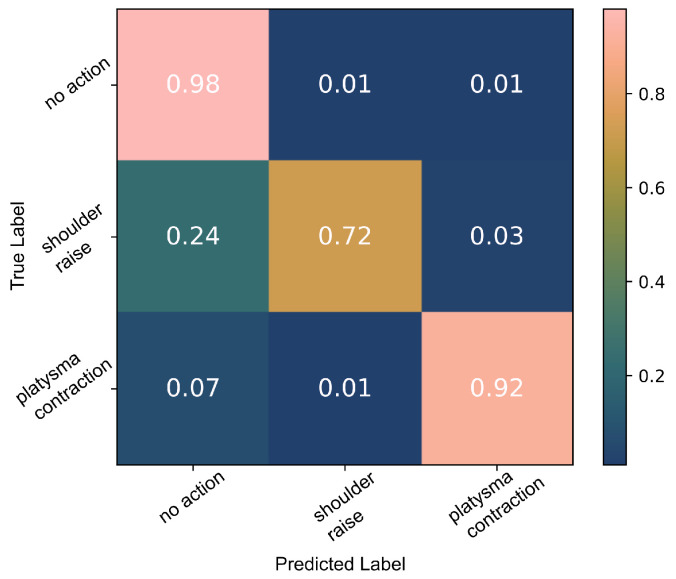
Decision tree classification results of the platysma sensor data captured with Participant2 during *shoulder raise* and *platysma contraction*, on a single day.

**Figure 12:**
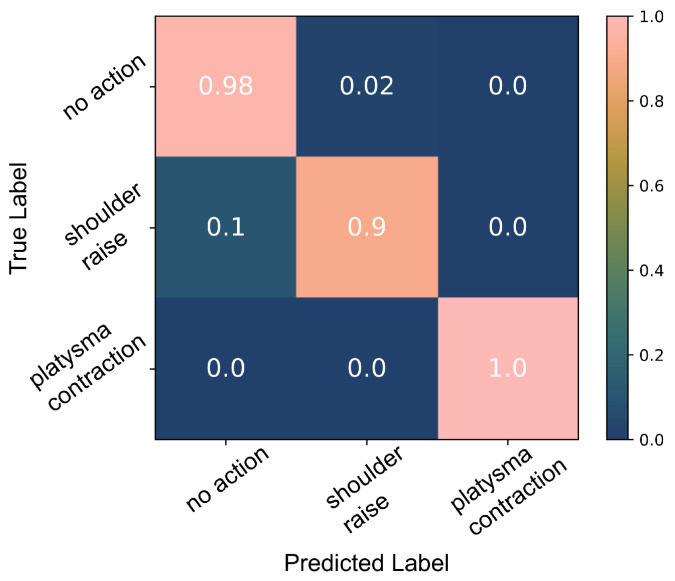
Classification results of the platysma sensor data using the thresholding algorithm.

**Figure 13:**
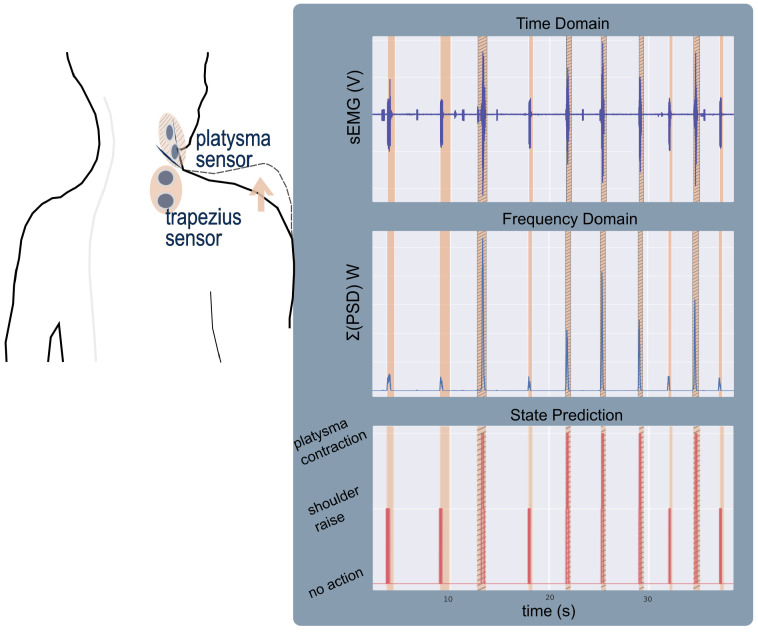
From top to bottom: The filtered EMG readings, the power of the signal and the state-prediction results from a sample of Participant2 data. The shaded regions represent the event occurrences; the hatched shaded regions indicate a *platysma contraction* and the non-hatched ones a *shoulder raise*.

The decision tree algorithm cannot perform well on the limited number of data available; the confusion matrix in Figure 11 demonstrates how only 72% of the shoulder raise data were correctly classified. Moreover, the performance was very inconsistent in every run of the algorithm. This is probably due to the inability of the algorithm to generate a model that captures the diversity of the data properly due to the limited number of data available. However, due to the clear separability of the data (demonstrated in Figure 2), the thresholding algorithm performs well when splitting the feature space into *no action*, *SR* and *PC*. A 97% accuracy was achieved as shown Figure 12. Figure 13 presents an example from the data of the platysma sensor and the separability of the two classes. The shaded regions on the plot indicate the *action windows*, the contraction of the trapezius (shaded areas) and platysma (hatched shaded areas) muscles.

## 4. Discussion

In this paper, we set out to demonstrate how a single sEMG sensor’s readings contains a vast amount of information when visualised in the frequency domain, both on the primary muscle being monitored as well as other sources in its proximity. We also showed how different movements of the same joint or actions that engage the same muscle give rise to different sEMG signatures. Such methodology demonstrates how one can reduce the number of necessary sEMG sensors in a system. In real-world scenarios, the platysma and trapezius muscles, which are currently being used for the AGILIS neuroprosthesis activation, are engaged during various activities of daily living (ADLs). It is crucial to distinguish between the sEMG signature of voluntary shoulder raise and platysma contraction, movements used to activate the AGILIS neuroprosthesis, and other actions that engage those same muscles, in order to develop reliable, robust, to noise and unknown actions, and practical HMIs. Distinctions between these need to be made in order to take all the state-of-the-art motion recognition interfaces from academic research to the user. Here, we take the first step in that direction as we look at different shoulder movements and attempt data classification with the readings of a single sensor.

### 4.1. Binary Classification

#### 4.1.1. Able-Bodied Participants

During the able-bodied participant experiments, the analysis of the signal in the frequency domain exhibited clear indications of muscle contraction. The data recorded by one sEMG channel captured the signatures of different movements. Despite each iteration of the movements being performed at variable strength levels and in a relaxed manner, we were able to consistently classify *shoulder raise* (*SR*) using a white-box algorithm with accuracies that match the state-of-the-art. *SR* was classified with 99% accuracy (Figure 4). The robustness of the model was tested when its performance was verified against both known (*SR*, *SF* and *SB*) and unknown data, *shoulder raise-and-hold* (*SRH*) and *object raise* (*OR*), with a 92% accuracy. As would be expected, *SRH* actions triggered some false positives. However, despite *SR* and *SRH* having the same agonists, their sEMG signatures are different, and therefore separable. To start with, the participants were, in general, unconsciously using a lower momentum when they anticipated stopping at the apex of the movement, i.e., during *SRH*. This means that there is a higher muscle fibre recruitment during the first part of the *SR* movement and thus the power of the signal is higher. Additionally, holding the shoulder up during *SRH* involves isometric contraction of the muscles and is something that does not take place during *SR*. As a result, the majority of *SRH* were correctly classified as *no action* instead of *SR* using the decision tree model.

The thresholding algorithm also performed well in this binary classification of the same data. The median of the *action* training data that was used as the threshold was appropriate in the able-bodied participant data as they varied the momentum and range of the movements performed causing a high variability in the data. This classification algorithm however did not have the same ability to distinguish between *SR* and *SRH*. The overwhelming majority of the false positives, when all data were used, as seen in Figure 6, were a result of *SRH*. This was not surprising as the two movements have the same agonists. It can be speculated that the reason why the decision tree performs better is because of its multilayered structure. A more complex architecture is also better suited to classify *SR* when the motion is performed with highly variable forces/moments as is the case in the experiment performed with able-bodied participants.

#### 4.1.2. Participants with Tetraplegia

Figure 8 demonstrates how, in the frequency domain, we are able to detect weak trapezius muscle contractions during the *SR* attempts and distinguish them from the noise with a low signal-to-noise ratio (SNR). For signals with low SNR, Figure 7, visualising the frequency domain features, shown in Figure 8b, can be very useful in identifying muscle contractions. There is a linear separation between the contraction and non-contraction data points and thus thresholding performs well on these data. All shoulder raise attempts were correctly identified in the two sets of data acquired with Participant1. For the same reason, we were able to detect *SR* in Participant2’s data using the trapezius muscle sensor and platysma contraction *PC* using the platysma sensor. A decision tree classifier could not perform as well as, in its attempts to find the best split for the data, it is not able to consistently capture the diversity in these data due to the limited number of data as well as the labelling inaccuracies, in particular with low SNR data. A single conditional statement, implement in the thresholding algorithm, is able to more accurately model the data compared to decision trees which are multilayered conditional statements and can capture higher complexity data.

### 4.2. Multi-Class Classification

#### 4.2.1. Able-Bodied Participants: Shoulder Movement

Using the able-bodied participant data, we trained the algorithm to classify the three shoulder movements, *SR*, *SF* and *SB*, using all data achieving a 94% accuracy, Figure 9. There were a few false positives (<7%) that could be attributed to labelling inaccuracies and the misclassification of *SRH* as *SR*. *SR* was easier to classify than the other shoulder movements; 98% of *SR* data were correctly classified in contrast to 92% and 94% for *shoulder forward* (*SF*) and *shoulder backward* (*SB*) movements, respectively. This is to be expected as the sensor used was located on top of the trapezius descendens (upper) muscle which is the primary muscle engaged during *SR*. The main protraction (*SF*) agonist muscles are the serratus anterior and pectoralis muscles. During shoulder retraction (*SB*), the main agonists are the middle trapezius and rhomboid muscles which are located in close proximity to the upper trapezius muscle but in the deeper layers. The signals generated by these nearby muscles can be detected over the trapezius descendens (upper) muscle as a result of crosstalk. Hence, the classification results of the *SB* movements were consistently higher despite having some *SR* false positives due to the same reasons. These observations are further supported by the SNR which was overall highest during shoulder raise movements and lowest during shoulder forward movements. For example, in one participant’s data, the SNR during shoulder raise was in the range of 5 dB to 7 dB, while during shoulder forward actions it was calculated at 2 dB to 3 dB.

A second decision tree model was trained using solely the *SF*, *SB* and *SF* data and the 10-fold cross-validation results are presented in Figure 10. Less than 1% of *no action* data points were classified as *SR* (false positives). During each cross-validation cycle, we tested the trained model against all data, including the ”unknown” *SRH* and *OR* data and a 90% accuracy was achieved. About 3% of the *no action* data were wrongly classified as *SR*, the majority of which were *SRH*. In addition to that, due to the engagement of the pectoralis muscle during *OR*, 2% of the *OR* data were falsely classified as *SF*, since the muscle also engages during shoulder protraction. *SF* is the most difficult to detect as its agonists are further away from the sensor placed on the upper trapezius muscle compared to *SR* and *SB*.

The performance of this system is on par with classification accuracy in a similar shoulder movement classification study where multiple sEMG sensors and convolutional neural networks were used [17], as well as other state-of-the-art classification systems [40]. As hypothesised, the trapezius muscle’s activity has a different sEMG signature depending on the movement being performed even due to the engagement of different muscles. These results demonstrate the robustness and reliability of this single-sensor, FD-feature, white-box motion recognition system. We are able to create an individualised motion recognition system. Although factors such as adipose tissue and skin permittivity may affect the sEMG readings, there were not any participant cases where the algorithm did not perform well. Further studies would have to be performed to determine the effect of electrode positioning on this frequency domain analysis and classification. It is worth noting that no fatigue is expected to have occurred as each participant trial was short, 1 min for each movement, with resting time in between, and it was requested that the participants perform the movements in a relaxed manner.

#### 4.2.2. Participants with Tetraplegia: PC and SR

There was evidence of *SR* movements in the platysma sensor sEMG signal. We therefore explored the possibility of identifying more than one body part’s activity with a single sensor, in particular, *SR* and *PC*, both of which have been used for the activation of the neuroprosthesis. The results of the decision tree classifier were inconsistent every time the classifier was trained and tested during cross-validation and did not have comparable accuracies as with the able-bodied participant data due to the limited number of samples and labelling inaccuracies. However, by applying the thresholding classification algorithm, we were able to better classify *PC* and *SR* actions. Using the power of the platysma sensor, we performed a three-way classification (*SR*/*PC*/*no action*), Figure 13, with a 95% accuracy as shown in Figure 12; these results are on par with published results of multi-sensor systems [40]. It is important to note that the data were classified for each day separately due to skin permittivity and sensor placement differences that affected the SNR.

Single-channel sEMG control could simplify a system or provide contingencies in more complex systems. Furthermore, features from proximal sensors can be used in conjunction to the sensor targeted at a specific muscle to determine the muscles activation or movement being attempted. For example, the platysma muscle’s data could be used to improve the reliability of a classification system. We have demonstrated that the sEMG sensor placed on the platysma muscle contains information on both the platysma muscle contraction and the shoulder raise. This is not true for the trapezius sensor data though; there are no clear signs of the platysma contractions. The reason why this happens has not been studied yet. The measurement changes can be explained by the passive movement of the platysma muscle, whose length may change during shoulder raise actions. Muscle synergies may also contribute to the potential difference changes measured by the platysma sensor. As the participants were attempting to raise their shoulder, they were inadvertently contracting the platysma muscle as well; this is especially prominent with individuals who have limited control of those muscles. Another hypothesis is that the signal is conducted through the body and a single sensor may detect activity from multiple sources. This is more probable to happen during a trapezius muscle contraction rather than a platysma muscle contraction as the trapezius muscle is larger and thus the attenuation of the signal is more probable. Picking a proximal signal’s attenuation is also dependent on the orientation of the sensor. Any of the above hypotheses could be true when *PC* and *shoulder raise* are performed.

## 5. Conclusions

In this paper, we have demonstrated how single-sEMG sensor systems can be used to recognise multiple movements. We used white-box algorithms to find a compromise between parsimony and robustness in order to develop systems that can be used outside the lab. With one sensor placed on the platysma muscle, shoulder raise and platysma contraction were successfully classified. We have shown that the number of sEMG electrodes may be reduced under certain circumstances, when desirable. In the frequency-domain representation of an sEMG signal, there could indeed be distinct information on the activation of two different muscles or movements of two separate joints. The white-box algorithms used may have to be adjusted to the individual and tuning might be necessary each day given the nature of sEMG. However, learning techniques could potentially be used to automatically make those calibrations. Taking advantage of such knowledge can lead to simpler and less cumbersome systems or make current systems more robust; in the case of a sensor failure, a lower number of sensors can be used to continue providing a reliable, accurate control input. What is more, the algorithm simplicity and transparency, and the reliability of a system such as the one presented here, could also lead to a safer, more reliable control of neuroprostheses.

## Figures and Tables

**Table 1:** This table presents the total amount of data used for classification for each able-bodied participant and each of the two participants with tetraplegia.

Participant	Action	Samples (k)	Action Occurrences
able-bodied individual	no action	2000	N/A
	shoulder raise	96	12
	shoulder forward	96	12
	shoulder backward	96	12
	shoulder raise-hold	120	12
	object raise	240	12
tetraplegic (P1)	no action	142	N/A
	shoulder raise	18	9
tetraplegic (P2)	no action	344	N/A
	shoulder raise	15	15
	platysma contraction	15	15

## Data Availability

Data sharing is not applicable to this article.

## Abbreviations

The following abbreviations are used in this manuscript:

SR	Shoulder raise (shoulder elevation)
SF	Shoulder forward (shoulder protraction)
SB	Shoulder backward (shoulder retraction)
SRH	Shoulder raise-hold
OR	Object raise
PC	Platysma contraction
